# Supplementary figures and images for: Sediment microbial taxonomic and functional diversity in a natural salinity gradient challenge Remane’s “species minimum” concept
Source: PeerJ. 2017 Oct 13;5:e3687. doi: 10.7717/peerj.3687 (PMC5642246; doi:10.7717/peerj.3687)

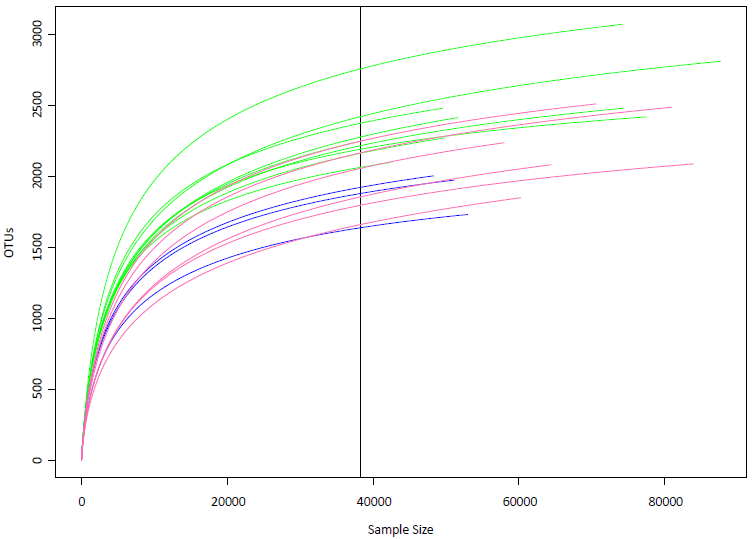

Supplement: Figure S1 — The vertical line (raremax =38,209) is the smallest number of individuals per sample to extrapolate the expected number of OTUs if all other samples had only that number of individuals. Rarefaction estimated the expected OTU richness in random subsamples of the community, with each subsample having 38,209 individuals. Blue: samples from Kalamitsi station. Pink: samples from Logarou stations. Green: samples from Arachthos stations. [file peerj-05-3687-s001.png]
