# Supplementary material for: Sediment microbial taxonomic and functional diversity in a natural salinity gradient challenge Remane’s “species minimum” concept
Source: PeerJ. 2017 Oct 13;5:e3687. doi: 10.7717/peerj.3687 (PMC5642246; doi:10.7717/peerj.3687)
Supplement: Table S1 — R, River; L, Lagoon; S, Sea; AR, Arachthos; ARO, Arachthos Neochori; ARDelta, Arachthos Delta; LOin, Logarou station inside the lagoon; LOout, Logarou station in the channel connecting the lagoon to the gulf; Kal, Kalamitsi; A, B, C, replicate samples. a: initial number of paired-end reads; b, number of paired-end reads after the quality trimming; c, number of paired-end reads with error-corrected bases; d, number of changed base during error-correction; e, number of bases that failed error-correction; f, total number of bases; g, final number of paired-end reads; h, number of overlapped reads. [file peerj-05-3687-s005.docx]

Supplementary Table 1: The results of the processing of the sequences. R: River. L: Lagoon. S: Sea. AR: Arachthos. ARO: Arachthos Neochori. ARDelta: Arachthos Delta. LOin: Logarou station inside the lagoon. LOout: Logarou station in the channel connecting the lagoon to the gulf. Kal: Kalamitsi. A, B, C: replicate samples. a: initial number of read pairs. b: number of read pairs after the quality trimming. c: number of read pairs with error-corrected bases. d: number of changed base during error-correction. e: number of bases that failed error-correction. f: total number of bases. g: final number of read pairs. h: number of overlapped reads.

| **Α/Α** | **Sample libraries** | **a** | **b** | **c** | **d** | **e** | **f** | **g** | **h** |
| --- | --- | --- | --- | --- | --- | --- | --- | --- | --- |
| **1** | R_AR_A | 97,520 | 97,257 | 103,854 | 119,220 | 17,195,293 | 49,005,323 | 97,140 | 85,492 |
| **2** | R_AR_Β | 157,246 | 156,744 | 150,418 | 175,130 | 22,017,363 | 79,182,431 | 156,539 | 138,330 |
| **3** | R_AR_C | 167,487 | 166,853 | 157,004 | 173,927 | 22,205,133 | 83,982,212 | 166,584 | 147,668 |
| **4** | R_ARO_A | 124,486 | 124,133 | 127,234 | 141,985 | 19,113,504 | 62,298,698 | 123,994 | 108,362 |
| **5** | R_ARO_Β | 148,083 | 147,533 | 142,035 | 158,385 | 20,558,495 | 74,288,330 | 147,315 | 130,082 |
| **6** | R_ARO_C | 142,040 | 141,410 | 138,108 | 156,909 | 20,503,832 | 71,090,930 | 141,171 | 124,829 |
| **7** | R_ARDelta_A | 99,760 | 99,117 | 127,995 | 144,962 | 22,421,254 | 48,221,228 | 98,852 | 83,896 |
| **8** | R_ARDelta_Β | 120,241 | 119,547 | 150,052 | 172,265 | 26,063,598 | 58,281,725 | 119,225 | 100,555 |
| **9** | R_ARDelta_C | 94,144 | 93,268 | 119,390 | 134,307 | 21,794,657 | 45,218,135 | 92,902 | 78,631 |
| **10** | L_LOin_A | 130,200 | 129,690 | 140,728 | 162,373 | 22,040,685 | 65,001,568 | 129,483 | 113,625 |
| **11** | L_LOin_B | 108,906 | 108,537 | 107,440 | 122,286 | 15,054,224 | 54,614,349 | 108,375 | 95,393 |
| **12** | L_LOin_C | 110,955 | 110,433 | 114,615 | 130,986 | 17,301,282 | 55,378,030 | 110,234 | 97,101 |
| **13** | L_LOout_A | 96,457 | 96,104 | 96,991 | 108,608 | 14,511,935 | 48,753,439 | 95,971 | 85,503 |
| **14** | L_LOout_B | 144,529 | 144,022 | 155,890 | 183,333 | 24,598,512 | 71,687,911 | 143,792 | 124,088 |
| **15** | L_LOout_C | 139,149 | 138,383 | 131,454 | 150,722 | 18,309,051 | 69,390,494 | 138,091 | 121,691 |
| **16** | S_Kal_A | 95,387 | 94,933 | 106,728 | 118,775 | 16,034,525 | 47,487,795 | 94,773 | 83,828 |
| **17** | S_Kal_B | 100,113 | 99,642 | 108,884 | 123,905 | 17,366,619 | 49,583,169 | 99,461 | 86,788 |
| **18** | S_Kal_C | 100,822 | 100,148 | 112,123 | 127,559 | 17,680,003 | 49,756,703 | 99,846 | 87,638 |
| **Sum** | |  |  |  |  |  |  |  | **1,893,500** |
