# Supplementary material for: Sediment microbial taxonomic and functional diversity in a natural salinity gradient challenge Remane’s “species minimum” concept
Source: PeerJ. 2017 Oct 13;5:e3687. doi: 10.7717/peerj.3687 (PMC5642246; doi:10.7717/peerj.3687)
Supplement: Table S2 — OTUs: total number of OTUs. N: total microbial relative abundance values. H’, Shannon-Wiener; J’, Pielou’s evenness; d, Margalef’s species richness; ACE, Abundance Coverage Estimator; *: p < 0.05. **: p < 0.01. n.s., not significant. [file peerj-05-3687-s006.docx]

Supplementary Table 2: The Chi-Square values of the Kruskal-Wallis tests for the diversity indices between the locations and the habitats. OTUs: total number of OTUs. N: total microbial relative abundance values. H΄: Shannon-Wiener. J΄: Pielou's evenness. d: Margalef’s species richness. ACE: Abundance Coverage Estimator. *: p < 0.05. **: p < 0.01. n.s.: not significant.

|  | **OTUs** | **N** | **d** | **J΄** | **H΄(ln)** | **Chao-1** | **ACE** |
| --- | --- | --- | --- | --- | --- | --- | --- |
| **Location** | n.s. | n.s. | 11.129 * | 13.28 * | 13.767 * | n.s. | n.s. |
| **Habitat** | 7.596 * | n.s. | 8.425 * | 13.177 ** | 12.966 ** | 7.799 * | 7.583 * |
