# Supplementary material for: Sediment microbial taxonomic and functional diversity in a natural salinity gradient challenge Remane’s “species minimum” concept
Source: PeerJ. 2017 Oct 13;5:e3687. doi: 10.7717/peerj.3687 (PMC5642246; doi:10.7717/peerj.3687)
Supplement: Table S3 — The values of the Mann-Whitney U tests used for the post-hoc pairwise significant comparisons, after the Bonferroni correction. OTUs, total number of OTUs; H’, Shannon-Wiener; J’, Pielou’s evenness; d, Margalef’s species richness; ACE, Abundance Coverage Estimator; AR, Arachthos; ARO, Arachthos Neochori; ARDelta, Arachthos Delta; LOin, Logarou station inside the lagoon; LOout, Logarou station in the channel connecting the lagoon to the gulf; Kal, Kalamitsi; *, p < 0.05 before the Bonferroni correction; **, p < 0.017 in the case of the habitats; n.s., not significant. [file peerj-05-3687-s007.docx]

Supplementary Table 3: The values of the Mann-Whitney U tests used for the *post-hoc* pairwise significant comparisons, after the Bonferroni correction. OTUs: total number of OTUs. H΄: Shannon-Wiener. J΄: Pielou's evenness. d: Margalef’s species richness. ACE: Abundance Coverage Estimator. AR: Arachthos. ARO: Arachthos Neochori. ARDelta: Arachthos Delta. LOin: Logarou station inside the lagoon. LOout: Logarou station in the channel connecting the lagoon to the gulf. Kal: Kalamitsi. *: p < 0.05 before the Bonferroni correction. **: p < 0.017 in the case of the habitats. n.s.: not significant.

|  |  | **OTUs** | **d** | **J΄** | **H΄(ln)** | **Chao-1** | **ACE** |
| --- | --- | --- | --- | --- | --- | --- | --- |
| **Location** | AR *vs* LOin | n.s. | n.s. | 3.971 * | n.s. | n.s. | n.s. |
|  | AR *vs* LOout | n.s. | n.s. | 3.971 * | n.s. | n.s. | n.s. |
|  | AR *vs* Kal | n.s. | n.s. | 4.091 * | n.s. | n.s. | n.s. |
|  | ARO *vs* LOin | n.s. | n.s. | 3.971 * | 3.971 * | n.s. | n.s. |
|  | ARO *vs* LOout | n.s. | n.s. | 3.971 * | 3.971 * | n.s. | n.s. |
|  | ARO *vs* Kal | n.s. | n.s. | n.s. | 3.971 * | n.s. | n.s. |
|  | ARDelta *vs* LOin | n.s. | n.s. | 3.971 * | n.s. | n.s. | n.s. |
|  | ARDelta *vs* LOout | n.s. | n.s. | 3.971 * | n.s. | n.s. | n.s. |
|  | ARDelta *vs* Kal | n.s. | n.s. | 4.091 * | n.s. | n.s. | n.s. |
| **Habitat** | River *vs* Lagoon | n.s. | n.s. | 10.366 ** | 10.198 ** | n.s. | n.s. |
|  | River *vs* Sea | 6.231 ** | 6.231 ** | 5.658 * | 6.319 ** | 6.231 ** | 6.231 ** |
|  | Lagoon *vs* Sea | n.s. | n.s. | n.s. | n.s. | 4.267 * | 4.267 * |
