# Supplementary material for: Sediment microbial taxonomic and functional diversity in a natural salinity gradient challenge Remane’s “species minimum” concept
Source: PeerJ. 2017 Oct 13;5:e3687. doi: 10.7717/peerj.3687 (PMC5642246; doi:10.7717/peerj.3687)
Supplement: Table S5 — The fraction of unexplained taxonomic units per sample and averaged per habitat, as it was derived during the functional profiling using UProC KEGG Ortholog reference profiles in long read mode. FTU, fraction of unexplained taxonomic units; R, River; L, Lagoon; S, Sea; AR, Arachthos; ARO, Arachthos Neochori; ARDelta, Arachthos Delta; LOin, Logarou station inside the lagoon; LOout, Logarou station in the channel connecting the lagoon to the gulf; Kal, Kalamitsi; A, B, C, replicate samples. [file peerj-05-3687-s009.docx]

Supplementary Table 5: The fraction of unexplained taxonomic units per sample and averaged per habitat, as it was derived during the functional profiling using UProC KEGG Ortholog reference profiles in long read mode. FTU: fraction of unexplained taxonomic units. R: River. L: Lagoon. S: Sea. AR: Arachthos. ARO: Arachthos Neochori. ARDelta: Arachthos Delta. LOin: Logarou station inside the lagoon. LOout: Logarou station in the channel connecting the lagoon to the gulf. Kal: Kalamitsi. A, B, C: replicate samples.

| **Samples** | **FTU (%)** | **Average FTU per location (%)** | **Average FTU per habitat (%)** |
| --- | --- | --- | --- |
| R_AR_A | 54.68 | 50.93 | 56.85 |
| R_AR_Β | 51.49 |  |  |
| R_AR_C | 46.61 |  |  |
| R_ARO_A | 49.98 | 48.29 |  |
| R_ARO_Β | 47.68 |  |  |
| R_ARO_C | 47.21 |  |  |
| R_ARDelta_A | 76.16 | 71.32 |  |
| R_ARDelta_Β | 69.64 |  |  |
| R_ARDelta_C | 68.17 |  |  |
| L_LOin_A | 67.68 | 66.95 | 67.51 |
| L_LOin_B | 67.94 |  |  |
| L_LOin_C | 65.22 |  |  |
| L_LOout_A | 75.18 | 68.06 |  |
| L_LOout_B | 66.04 |  |  |
| L_LOout_C | 62.97 |  |  |
| S_Kal_A | 37.9 | 38.91 | 38.91 |
| S_Kal_B | 39.92 |  |  |
| S_Kal_C | 38.92 |  |  |
