# Supplementary material for: Sediment microbial taxonomic and functional diversity in a natural salinity gradient challenge Remane’s “species minimum” concept
Source: PeerJ. 2017 Oct 13;5:e3687. doi: 10.7717/peerj.3687 (PMC5642246; doi:10.7717/peerj.3687)
Supplement: Table S7 — The linear regression equations for the average number of OTUs per taxonomic group, as well as the average number of OTUs irrespective of taxonomic group, at the different salinities. *, p < 0.05; **, p < 0.01; n.s., not significant; SW, p-value of Shapiro–Wilk statistic to test for normality in regression residuals (critical value ( a = 0.05) for Shapiro–Wilk statistic when n = 6 is 0.7923). [file peerj-05-3687-s011.docx]

Supplementary Table 7: The linear regression equations for the average number of OTUs per taxonomic group, as well as the average number of OTUs irrespective of taxonomic group, at the different salinities. *: p < 0.05. **: p < 0.01. n.s.: not significant. SW: p-value of Shapiro–Wilk statistic to test for normality in regression residuals (critical value (a = 0.05) for Shapiro–Wilk statistic when n = 6 is 0.7923).

|  | **Taxonomic group** | **Linear equation** | **R^2^** |  | **SW** |
| --- | --- | --- | --- | --- | --- |
|  | All OTUs | y = -13.322x + 2588.4 | 0.6719 | * | 0.3366 |
| Archaea | Crenarchaeota | y = 0.3862x + 6.7355 | 0.552 | n.s. |  |
|  | Euryarchaeota | y = 1.7602x + 42.142 | 0.3178 | n.s. |  |
|  | Unclassified Archaea | y = 1.3505x + 24.152 | 0.4197 | n.s. |  |
| Bacteria | Acidobacteria | y = -1.1499x + 118.77 | 0.51 | n.s. |  |
|  | Actinobacteria | y = -2.5228x + 127.28 | 0.8638 | ** | 0.6748 |
|  | Bacteroidetes | y = -5.9149x + 483.59 | 0.7537 | * | 0.9983 |
|  | Chlorobi | y = -0.2292x + 19.104 | 0.7611 | * | 0.8545 |
|  | Chloroflexi | y = -1.2697x + 120.66 | 0.5828 | n.s. |  |
|  | Cyanobacteria/Chloroplast | y = 0.283x + 23.533 | 0.1213 | n.s. |  |
|  | Firmicutes | y = -0.4473x + 36.236 | 0.7097 | * | 0.7609 |
|  | OD1 | y = -0.7155x + 40.82 | 0.8038 | * | 0.8457 |
|  | Planctomycetes | y = -0.2028x + 91.737 | 0.0124 | n.s. |  |
|  | Proteobacteria | y = -6.9127x + 728.14 | 0.6514 | n.s. |  |
|  | TM7 | y = -0.7108x + 33.437 | 0.9814 | ** | 0.8424 |
|  | Unclassified Bacteria | y = 3.824x + 509.41 | 0.2806 | n.s. |  |
|  | Verrucomicrobia | y = -1.5924x + 100.18 | 0.8384 | * | 0.1759 |
|  | Other | y = 0.7423x + 82.471 | 0.0812 | n.s. |  |
| Bacteroidetes | Bacteroidetes incertae sedis | y = -0.6164x + 22.668 | 0.971 | ** | 0.8067 |
|  | Bacteroidia | y = -0.3285x + 12.704 | 0.9297 | ** | 0.6491 |
|  | Flavobacteria | y = -0.3244x + 74.333 | 0.1095 | n.s. |  |
|  | Sphingobacteria | y = -1.5084x + 121.92 | 0.5637 | n.s. |  |
|  | Unclassified Bacteroidetes | y = -3.1372x + 251.96 | 0.7839 | * | 0.6318 |
| Proteobacteria | Alphaproteobacteria | y = -2.8399x + 223.18 | 0.655 | n.s. |  |
|  | Betaproteobacteria | y = -2.8083x + 100.92 | 0.939 | ** | 0.308 |
|  | Gammaproteobacteria | y = -0.4691x + 185 | 0.0823 | n.s. |  |
|  | Deltaproteobacteria | y = -0.7239x + 190.73 | 0.2751 | n.s. |  |
|  | Epsilonproteobacteria | y = -0.1367x + 11.211 | 0.7642 | * | 0.5321 |
|  | Unclassified Proteobacteria | y = 0.0651x + 17.105 | 0.0245 | n.s. |  |
